# Post-ESWL urinary osteopontin level and ion-activity product of calcium oxalate are associated with stone recurrence after 5-year follow-up

**DOI:** 10.1186/s12894-025-01791-x

**Published:** 2025-04-24

**Authors:** Chia-Min Liu, Chan-Jung Liu, Ze-Hong Lu, Ho-Shiang Huang

**Affiliations:** 1 Department of Urology, National Cheng Kung University Hospital, No.138, Sheng-Li Road, Tainan City, Taiwan; 2 Department of Urology, College of Medicine, National Cheng Kung University, Tainan, Taiwan

**Keywords:** Biomarkers, Lithotripsy, Nephrolithiasis, Recurrence, Urolithiasis

## Abstract

**Background:**

Calcium oxalate (CaOx) is the predominant component in over 80% of kidney stones. The formation and retention of kidney stones involve multiple mechanisms, including overgrowth on Randall’s plaques—subepithelial calcium phosphate deposits in the renal papillae—and interactions between urinary crystals and renal tubular epithelial cells. Previous studies have shown that specific urinary proteins can either promote or inhibit CaOx crystal nucleation, growth, and retention. This study investigates changes in the urinary excretion of CaOx-related proteins before and after extracorporeal shock wave lithotripsy (ESWL), aiming to identify protein markers associated with stone formation and recurrence over a 5-year follow-up.

**Methods:**

This study enrolled 54 patients with renal or ureteral stones treated with ESWL and 13 healthy controls. 24-hour urine samples were collected preoperatively and at 2 and 4 weeks post-ESWL for biochemical analysis. CaOx-related proteins were measured semi-quantitatively in urine samples before and after ESWL, including nucleolin-related protein (NRP), nuclear pore complex p62 (NPC), hyaluronic acid (HA), SLC26A6, CXCR4, osteopontin (OPN), Tamm-Horsfall protein (THP) and Matrix Metalloproteinase (MMPs). Statistical analyses, including Pearson correlation and logistic regression, were performed to identify factors related to 5-year stone recurrence. Statistical significance was set at *p* < 0.05.

**Results:**

Before ESWL, stone patients exhibited significantly higher levels of NPC, HA, and CXCR4. All examined markers, except for Tamm-Horsfall protein, increased significantly at two weeks after ESWL. At four weeks post-EWSL, OPN and SLC26A6 remained higher. Correlations were observed between CXCR4, MMPs, stone size and ion-activity product of calcium oxalate (AP_CaOX_), a measure of urinary calcium oxalate supersaturation. Logistic regression identified urinary OPN and AP_CaOX_ at 4 weeks post-ESWL as significant factors influencing 5-year stone recurrence, with a recurrence rate of 48.4%.

**Conclusions:**

The persistence of certain urinary proteins after ESWL implied the risk of stone recurrence despite stone removal. The stone recurrence at 5 years was significantly associated with higher urinary OPN and higher AP_CaOX_ levels at four weeks post-ESWL, suggesting the potential predictive value of these markers.

**Trial registration:**

Approval was obtained from the ethics committee of National Taiwan University Hospital (National Taiwan University Hospital Ethics Committee approval: 200706054M).

## Background

Kidney stone formation is a multifactorial process, arising from interactions between glomerular filtrate constituents and the renal tubular epithelial cells [1]. Recurrence rates after passing a first calcium stone are 15% at one year, 35–40% at 5 years, and 50% at 10 years according to studies regarding the natural history of urinary stone disease [2, 3]. However, there is currently no effective clinical method to predict stone recurrence after intervention.

Calcium oxalate stones, the most common type of kidney stones, are thought to originate through multiple mechanisms. One well-characterized pathway involves the formation of Randall’s plaques—subepithelial calcium phosphate deposits in the renal papillae—which serve as a nidus for CaOx crystal overgrowth when the overlying epithelium is breached [4]. Alternatively, stones may form through direct crystal adhesion to injured renal tubular epithelial cells, especially under conditions of oxidative stress or altered tubular microenvironments. These distinct but potentially coexisting mechanisms underscore the complexity of stone pathogenesis and retention within the kidney.

Crystallization begins with nucleation, followed by crystal growth, aggregation, and ultimately adhesion to renal tubular surfaces [5]. As most urinary crystals are excreted during micturition, the adherence of crystals was considered to be the first event of retention [6]. While undisturbed tubular epithelial cells had little capacity for crystal attachment [7], disruptions in tight junctions during cellular proliferation or migration can enhance crystal affinity for calcium oxalate(CaOx) on apical membranes [7].

Over 80% of kidney stones are composed of CaOx [4], with the remaining 2 to 5% containing organic matrix, predominantly protein [8, 9]. Certain urinary proteins could either inhibit or promote crystal nucleation, growth and aggregation, affecting their attachment to kidney tubules lumens [8]. Several crystal-adhering molecules, including sialic acid [10], hyaluronic acid (HA) [11], chondroitin sulfate and heparin sulfate molecules, are believed to play a major role in crystal attachment to epithelial cells [6]. Nucleolin-related protein (NRP), found on the surface of cultured inner medullary collecting duct cells, selectively absorb CaOx and bind to membrane skeletal elements in a Ca-dependent manner [12].

The alterations in tubular epithelial cell surface molecules that could contribute to crystal retention including genetic modification, cell injury or drugs exposure [10]. Selvam et al. suggested that sub-lethal injury mediated by ischemia or oxalate induced lipid peroxidation reaction can expose concealed sialic acid residues, facilitating crystal adhesion [6]. Crystal adhesion-related molecules, including osteopontin (OPN) and Tomm-Horsfall glycoprotein (THP), increase during crystal formation [10, 13], regulating crystal nucleation, growth, aggregation and interaction with the tubular epithelium [14].

Sun et al. proposed that oxalate-specific binding molecules, like nuclear pore complex oxalate binding protein p62 (NPC) [15], possess crystal growth modulating activity similar to calcium-related binding proteins. These molecules potentially facilitate the movement of oxalate or CaOx across kidney tubule membranes, affecting secretion and detoxification pathways [11]. The kidney tubules express three members of the SLC26A family capable of oxalate transport [12], with *SLC26A6* knockout mice exhibiting hyperoxaluric and susceptibility to CaOx nephrolithiasis [13–15]. This suggests a potential role for the SLC26A6 transporter in reducing urinary oxalate excretion [12].

Increased leukocyte infiltration of renal interstitium, giant cells surrounding CaOx crystals, and interstitial fibrosis were found in the experimental hyperoxaluric kidney [16]. Stromal cell-derived factor-1 (SDF-1) and CXCR4 interaction triggers migration and chemotaxis of lymphocytes, monocytes, and neutrophils [17], with CXCR4 expression indicative of leukocyte infiltration [16, 17].

Matrix Metalloproteinases (MMPs), particularly MMP-2 and MMP-9, facilitate extracellular matrix (ECM) accumulation and may enhance CaOx crystal attachment to the injured basement membranes [18, 19]. MMPs are also potential regulators of cell migration and invasion [16, 17].

Most data on CaOx crystal formation proteins were collected from animal and cell line study. One clinical study reported that the variations in the abundance and electrophoretic mobility of specific urine proteins are associated with stone formation [20].

To investigate whether certain CaOx-related proteins exhibit association with nephrolithiasis, we conducted this prospective study aiming at assessing the alterations in the urinary excretion levels of CaOx-related proteins, including HA, NRP, NPC, SLC26A6, THP, OPN, CXCR4, MMP-2, and MMP-9, in CaOx stones patients before and after extracorporeal shock wave lithotripsy (ESWL). Correlations were made between urinary protein levels, multiple clinical parameters and the stone recurrence over a 5-year follow-up.

## Methods

### Study participants and data collection

The study (National Taiwan University Hospital Ethics Committee approval:200706054 M) enrolled patients with a single renal stone (diameter < 2 cm) or ureteral stone admitted to the Department of Urology at National Taiwan University Hospital for ESWL using a Siemens Lithostar^®^ Multiline (Siemens, Erlangen, Germany) lithotripter which were performed by one urologist, conducted from July 2007 to June 2008. Stone-free status was assessed at 3 months post-ESWL using kidney-ureter-bladder (KUB) radiography. Patients were considered stone-free if no residual stone fragments larger than 3 mm were detected; fragments ≤ 3 mm were considered clinically insignificant. Only patients achieving stone-free status based on this definition were included in the study, ensuring no residual stones were present at baseline.

Exclusion criteria including the history of hyperparathyroidism, other inflammatory or malignant disease, diabetes mellitus, heart disease, age < 20 years old, estimation glomerular filtrating rate (eGFR) less than 15 ml/min/1.73m^2^ [5], 24-hour urinary creatinine levels < 15 mg/Kg per day(men) or < 10 mg/Kg per day(women). Patients receiving medications affecting stone metabolism, as well as those with congenital urinary tract anomalies or significant urinary obstruction requiring intervention, were excluded. Mild hydronephrosis secondary to stones was allowed if it resolved post-ESWL without further intervention. Notably, no enrolled patients presented with urinary anomalies, so no exclusions were made on this basis.

Stone recurrence was evaluated with medical records from 2012 July to 2013 Feb (Ethics Committee Approval: 201303105RINC). Stone recurrence was defined as the appearance of newly developed stones identified by imaging studies (KUB, ultrasound, or CT) during the follow-up period, following confirmation of complete stone clearance (no residual fragments > 3 mm) at 3 months post-ESWL. Of 70 enrolled patients (57 men, 13 women), 54 achieved stone-free after ESWL (41 men, 13 women). Stone fragments retrieved after ESWL were analyzed by infrared spectroscopy, which is routinely used in our institution for stone composition determination. However, due to the nature of ESWL—where fragments may be too small to collect or unnoticed by patients—stone analysis could not be performed for all cases.

Pre-operative 24-hour urine sample were collected, and urine volume recorded. Urine sample also underwent sediment and electrolyte analysis, with remainders frozen at -20 °C. Follow-up 24-hour urine samples were collected at 2 and 4-weeks post-ESWL.

The control group comprised 13 normal volunteers (mean age: 54.5 ± 3.5 years), free of urinary stone and exclusion criteria.

### Determination of urine biochemistry

Urine samples underwent centrifugation (2000 *g*, 10 min) for debris removal. The supernatants were analyzed for oxalate, calcium (Ca), magnesium (Mg), citrate, and tubular enzymes (αGST, πGST). Urinary oxalate and citrate levels were examined as previously described [5]. Urinary ion-activity product of CaOx(AP_CaOx_) was assessed using the formula by Tiselius et al. [19]. Quantitative solid phase enzyme immunoassays (Biotrin NEPHKIT-Pi for Human πGST and Biotrin Human αGST Enzymoimmunoassay, Biotrin International, Dublin, Ireland) were used to assess urinary levels of αGST and πGST. Urinary lipid peroxides were examined by measuring the malondialdehyde (MDA) with a BIOXYTECHMDA-586 kit (Oxis Research, Portland, Ore). All assays were conducted in duplicate.

### Determination of urinary levels of CaOx -associated proteins

Urinary protein expressions were examined by Western blot as our previous method [18]. Primary antibodies to HA, NRP, NPC, SLC26A6, THP, OPN, CXCR4, MMP-2, and MMP-9 were obtained from Santa Cruz Biotechnology (Santa Cruz, CA, USA). Urine supernatant (2 ml) was mixed with PBS containing protease inhibitor cocktail (1 mM pepstatin, 200 mM phenylmethylsulfonyl fluoride, 1 mM leupeptin, and 1mM ethylenediaminetetraacetic acid; Roche, Mannheim, Germany) and concentrated with build-in diaphragm vacuum pump (Model 5301, Eppendorf, Germany). Urinary protein concentrations were determined with Bio-Rad Dc protein assay kit (Hercules, CA, USA). Each preparation (120 µg of protein) was separated on SDS polyacrylamide gels, electrophoretically transferred onto PVDF membranes (Amersham, Buckingham, England, UK). After blocking for 1 h at room temperature with 5% milk in Tris-buffered saline, the membrane was incubated overnight with appropriate polyclonal antibodies in blocking solution. After washed in Tris-buffered saline containing 0.1% Tween-20 and incubation with HRP-conjugated IgG antibodies, band densities were determined semiquantitatively on a densitometer with an image analytic system (Alpha Innotech, San Leandro, CA).

### Data analysis

Continuous variables were expressed as mean ± standard deviation and compared using Student’s t-test or ANOVA where appropriate. The Duncan’s multiple range test was used exclusively to compare urinary biomarker levels at different time points (pre-ESWL, 2 weeks, and 4 weeks post-ESWL) to identify significant differences between paired groups.

The Pearson product-moment correlation was used to determine the correlation coefficient (r value) between urinary proteins (HA, NPC, NRP, SLC26A6, OPN, THP, MMP2, MMP9, CXCR4) and clinical parameters (tubular enzymuria, stone size, AP_CaOx_, and urinary MDA). Temporal changes among pre-ESWL, 2-week post-EWSL, and 4-week post-EWSL periods were compared using generalized linear model (GLM) for repeated measurements. Logistic regression was used for univariate and multivariate analyses to identify factors associated with stone recurrence. Statistical analysis was performed using SPSS, v16 (SPSS, Inc., Chicago, IL, USA) with *p* < 0.05 indicating significance.

## Results

### Urinary profiles in ESWL-Treated stone patients versus controls

Table 1 compares basic characteristics and urinary proteins between ESWL-treated stones patients and controls, showing no difference in BMI and creatinine clearance. Stone patients exhibited significantly higher AP_CaOx_ and urinary oxalate levels, along with lower urinary citrate levels compared to controls. However, the levels did not change remarkably after CaOx stone removed. Stone patients also displayed elevated urinary tubular marker levels (α-GST and π-GST) and urinary lipid peroxide excretion (MDA) that persisted at 2 weeks and 4 weeks post-ESWL.

**Table 1:** Basic data and laboratory parameters of control and stone patients

	Control (*n* = 13)	Stone patients		
		Before ESWL (*n* = 54)	2 wk after ESWL (*n* = 44)	4 wk after ESWL (*n* = 37)
Age (years)	54.5 ± 3.8	53.8 ± 3.0		
BMI ((kg/m^2^)	23.6 ± 0.4	23.5 ± 0.7		
Stone size (mm)	0	9.1 ± 2.3*		
Urine pH	6.0 ± 0.1	6.2 ± 0.2	6.0 ± 0.2	6.1 ± 0.1
eGFR (ml/min/1.73 m²)	88.3 ± 4.7	87.6 ± 5.4	88.2 ± 5.6	85.1 ± 4.6
AP_CaOx_	2.4 ± 0.9	4.7 ± 1.1*	4.8 ± 1.1^#^	4.7 ± 1.1^+^
Oxalate (mg/24 h)	9.1 ± 1.8	40.4 ± 4.9*	47.8 ± 7.5^#^	44.6 ± 11.9^+^
Citric acid (mg/24 h)	365.8 ± 42.1	102.4 ± 20.4*	96.8 ± 13.1^#^	86.9 ± 16.9^+^
Urine protein (g/24 h)	0.09 ± 0.01	0.07 ± 0.02	0.07 ± 0.02	0.08 ± 0.01
αGST (µg/g Cr)	5.3 ± 0.8	28.4 ± 5.9*	26.3 ± 3.6^#^	25.7 ± 3.3^+^
πGST (µg /g Cr)	4.9 ± 0.6	22.8 ± 4.5*	19.1 ± 4.1^#^	20.7 ± 3.8^+^
MDA (µmol/g Cr)	11.7 ± 2.4	44.5 ± 2.3*	41.8 ± 2.2^#^	39.8 ± 2.4^+^

* *p* < 0.05 when compared with the controls;

# *p* < 0.05 when compared with stone patients before ESWL;

+ *p* < 0.05 when compared with stone patients 2 weeks after ESWL

Abbreviations: BMI, body mass index; ESWL, extracorporeal shock wave lithotripsy; APCaOx, ion-activity product of calcium oxalate; αGST, alpha-glutathione S-transferase; πGST, pi-glutathione S-transferase; MDA, malondialdehyde; Cr, creatinine

**Fig. 1:**
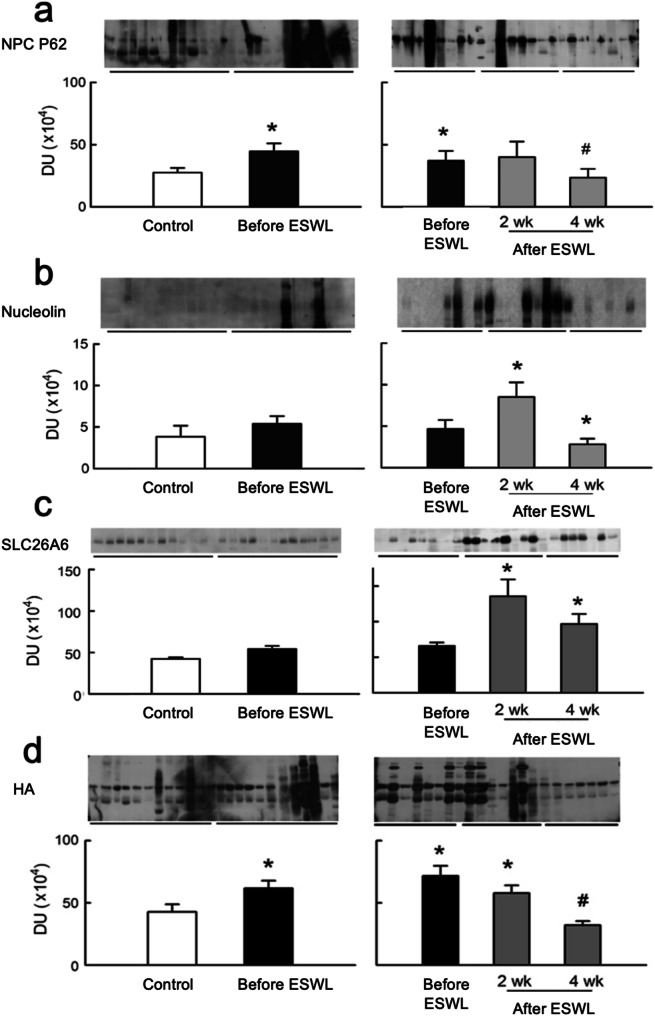
Changes in urinary expression of oxalate- and CaOx-related proteins. (**a**) NPC P62 (Nuclear pore complex oxalate binding protein p62, 62 kDa) in urine was significantly higher in pre-ESWL stone patients compared to the controls (**b**) Nucleolin (77 kDa) significantly increased at 2 weeks but notably decreased at 4 weeks. (**c**) SLC26A6 (90 kDa) significantly increased at 2 weeks but notably decreased at 4 weeks. (**d**) HA (Hyaluronic acid, 5.5 kDa) was significantly higher in pre-ESWL stone patients compared to the controls. HA significantly increased at 2 weeks but notably decreased at 4 weeks

Figure 1 illustrates changes in urinary expression of oxalate and CaOx related proteins. Pre-ESWL, stone patients exhibited significantly higher urinary levels of HA and NPC compared to controls. Post-ESWL, NRP, SLC26A6, and HA significantly increased at 2 weeks but notably decreased at 4 weeks. SLC26A6 remained higher at 4 weeks post-ESWL than pre-ESWL but lower than at 2 weeks post-ESWL in stone patients.

### Correlation analysis of urinary biomarkers in stone patients pre and post ESWL

Stone patients exhibited consistently positive correlations between urinary HA levels, tubular enzymuria, AP_CaOx_ and urinary MDA levels across all observed periods, including before ESWL, at 2-weeks, and at 4 weeks post-ESWL, respectively as shown in Table 2. Moreover, urinary NPC levels were negatively correlated with πGST at 2-week (*r* = -0.22) and 4-week (*r* = -0.13) post-ESWL. Comparing the temporal change of correlations between urinary NPC and clinical parameters revealed significant differences in urinary NPC vs. urinary πGST, and urinary NPC vs. urinary MDA (*p* < 0.05), indicating the event of stone free post-ESWL resulted in the negative association between urinary NPC with urinary πGST and urinary MDA over time.

**Table 2:** Comparison of Pre/Post-ESWL urinary enzymes with stone Size, APCaOx, and MDA

		Stone size	πGST	αGST	AP_CaOx_	MDA
**HA**	Pre-EWSL	*r* = 0.46†	*r* = 0.92†	*r* = 0.93†	*r* = 0.56†	*r* = 0.93†
	2-WK		*r* = 0.95†	*r* = 0.92†	*r* = 0.62†	*r* = 0.92†
	4-WK		*r* = 0.91†	*r* = 0.89†	*r* = 0.60†	*r* = 0.93†
	***p*** **value** §		*p* < 0.05*	*p* < 0.05*	*p* = 0.06	*p* = 0.21
**NPC**	Pre-EWSL	*r* = -0.08	*r* = 0.09	*r* = 0.27	*r* = 0.03	*r* = -0.03
	2-WK		*r* = -0.22	*r* = 0.14	*r* = 0.01	*r* = − 0.16
	4-WK		*r* = -0.13	*r* = 0.80	*r* = -0.17	*r* = − 0.06
	***p*** **value** §		*p* < 0.05*	*p* = 0.24	*p* = 0.85	*p* < 0.05*
**NRP**	Pre-EWSL	*r* = 0.12	*r* = 0.31	*r* = 0.47	*r* = 0.18	*r* = 0.22
	2-WK		*r* = 0.28	*r* = 0.47	*r* = 0.25	*r* = 0.22
	4-WK		*r* = 0.66†	*r* = 0.55	*r* = 0.21	*r* = -0.30
	***p*** **value** §		*p* = 0.87	*p* = 0.51	*p* = 0.13	*p* < 0.05*
**SLC26A6**	Pre-EWSL	*r* = 0.07	*r* = 0.02	*r* = 0.74†	*r* = 0.23	*r* = 0.74†
	2-WK		*r* = -0.14	*r* = 0.85†	*r* = 0.06	*r* = 0.85†
	4-WK		*r* = 0.13	*r* = 0.52†	*r* = 0.04	*r* = 0.53†
	***p*** **value** §		*p* = 0.92	*p* < 0.001*	*p* = 0.67	*p <* 0.05*
**OPN**	Pre-EWSL	*r* = -0.77†	*r* = -0.72†	*r* = -0.76†	*r* = 0.32	*r* = -0.81†
	2-WK		*r* = -0.54	*r* = -0.56	*r* = 0.40	*r* = -0.43
	4-WK		*r* = -0.83†	*r* = -0.66†	*r* = 0.31	*r* = -0.77†
	***p*** **value** §		*p <* 0.05*	*p* < 0.001*	*p* = 0.74	*p* < 0.05*
**THP**	Pre-EWSL	*r* = 0.06	*r* = 0.22	*r* = 0.14	*r* = 0.13	*r* = 0.14
	2-WK		*r* = 0.12	*r* = 0.15	*r* = 0.07	*r* = 0.15
	4-WK		*r* = 0.12	*r* = 0.12	*r* = 0.05	*r* = 0.13
	***p*** **value** §		*p* = 0.88	*p* = 0.95	*p* = 0.77	*p* = 0.82
**CXCR4**	Pre-EWSL	*r* = 0.88†	*r* = -0.13	*r* = -0.12	*r* = 0.85†	*r* = -0.13
	2-WK		*r* = 0.29	*r* = 0.17	*r* = 0.84†	*r* = 0.29
	4-WK		*r* = 0.28	*r* = 0.04	*r* = 0.72†	*r* = 0.28
	***p*** **value** §		*p* = 0.39	*p* = 0.09	*p* < 0.01*	*p* = 0.43
**MMP2**	Pre-EWSL	*r* = 0.27†	*r* = 0.048	*r* = 0.05	*r* = 0.13†	*r* = 0.05
	2-WK		*r* = 0.27	*r* = 0.26	*r* = 0.80†	*r* = 0.27
	4-WK		*r* = 0.39	*r* = -0.09	*r* = 0.54†	*r* = 0.29
	***p*** **value** §		*p* = 0.98	*p* = 0.09	*p* < 0.05*	*p* = 0.98
**MMP9**	Pre-EWSL	*r* = 0.28†	*r* = 0.05	*r* = 0.05	*r* = 0.13†	*r* = 0.05
	2-WK		*r* = 0.27	*r* = 0.26	*r* = 0.80†	*r* = 0.27
	4-WK		*r* = -0.29	*r* = -0.09	*r* = 0.54†	*r* = 0.29
	***p*** **value** §		*p* = 0.98	*p* = 0.09	*p* < 0.05*	*p* = 0.98

2 WK: 2 weeks post-ESWL

4 WK: 4 weeks post-ESWL

†: *p* value showed significance of the correlation coefficient (*r* value) between the variables examined at the examined period

§: *p* value examined by generalized linear model for repeated measurements among the all examined periods (* *p* < 0.05)

Abbreviations: ESWL, extracorporeal shock wave lithotripsy; APCaOx, ion-activity product of calcium oxalate; MDA, malondialdehyde; GST, glutathione S-transferase; HA, hyaluronic acid; NPC, nuclear pore complex p62; NRP, nucleolin-related protein; OPN, osteopontin; THP, Tamm-Horsfall protein; SLC26A6, solute carrier family 26 member 6 (oxalate transporter); CXCR4, C-X-C chemokine receptor type 4; MMP, matrix metalloproteinase; πGST, pi-glutathione S-transferase; αGST, alpha-glutathione S-transferase

Urinary NRP levels at 4 weeks post-EWSL showed significant positive correlations with urinary πGST (*r* = 0.66, *p* < 0.05) (Table 2). Temporal changes in correlations were significant for NRP vs. stone size and NRP vs. urinary MDA in stone patients.

For urinary SLC26A6, positive correlations were found between SLC26A6 vs. αGST (*r* = 0.74 for before ESWL, *r* = 0.85 for 2-week after ESWL, and *r* = 0.52 for 4-week after ESWL, *p* < 0.05) and SLC26A6 vs. urinary MDA (*r* = 0.74 for before ESWL, *r* = 0.85 for 2-week after ESWL, and *r* = 0.53 for 4-week after ESWL, *p* < 0.05). GLM analysis indicated significant temporal changes in correlations for SLC26A6 vs. αGST and SLC26A6 vs. urinary MDA in stone patients.

### OPN and THP pre and post ESWL: correlation analysis

**Fig. 2:**
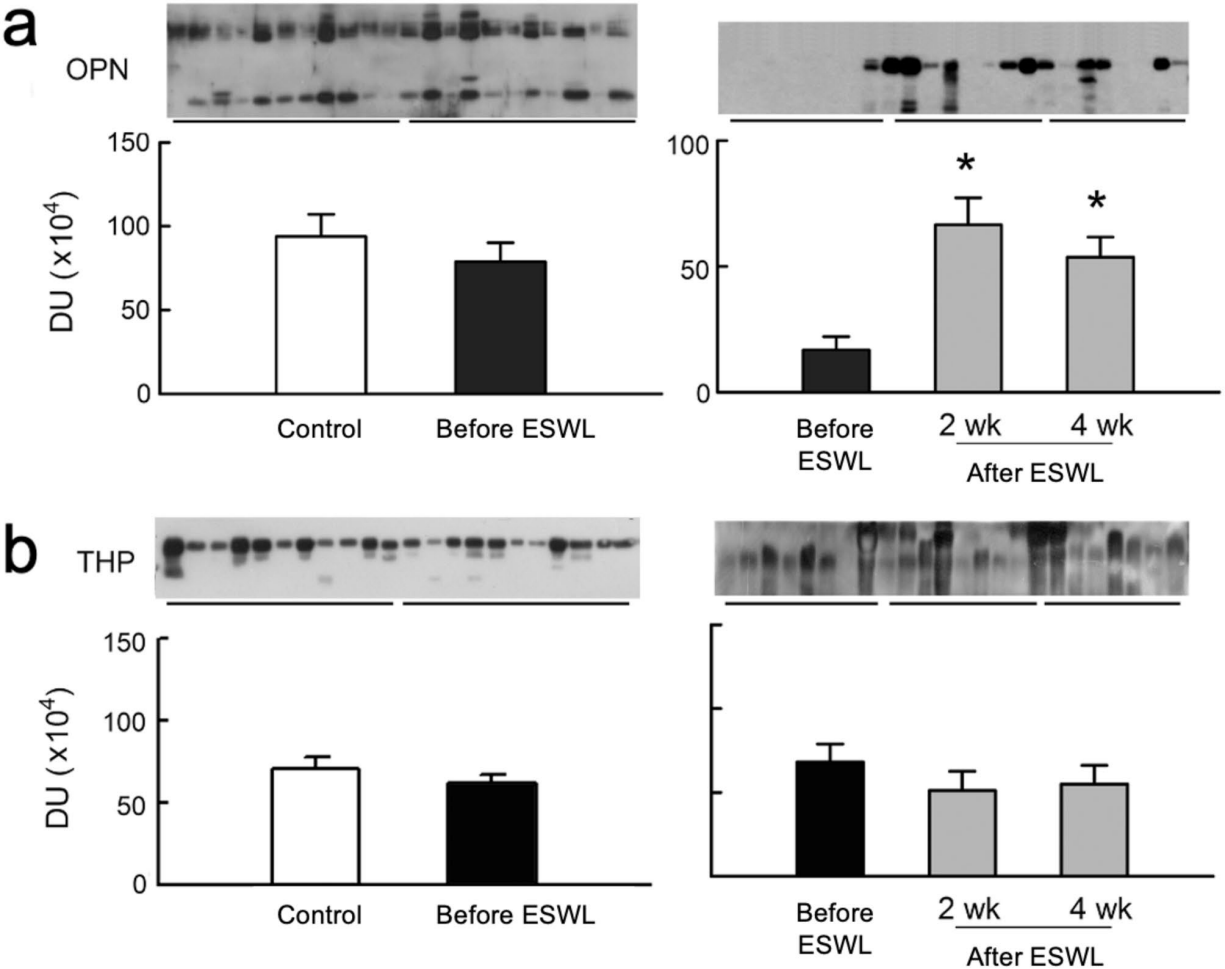
Changes in urinary expression of anticrystallization molecules. (**a**) Stone patients exhibited a significant increase in urinary OPN (Osteopontin, 44 kDa) levels at 2-week and 4-week post-ESWL in comparison to pre-ESWL levels and controls (**b**) Urinary THP(Tamm-Horsfall protein, 95 kDa) levels remained consistent across samples collected before ESWL and at 2-week and 4-week intervals post-ESWL in stone patients

Figure 2; Table 2 illustrate urinary expression of anticrystallization molecules and their correlations with urinary variables. Stone patients showed no significant difference in urinary OPN and THP levels compared to controls before ESWL. However, urinary OPN levels significantly increased at 2-week and 4-week post-ESWL, while THP levels remained consistent across pre-ESWL and post-ESWL samples in stone patients (Fig. 2). Negative correlations were observed between THP and πGST, αGST, and MDA before ESWL and at 4-week post-ESWL in stone patients, with no correlations found between urinary THP and the examined urinary variables (Table 2).

### Urinary levels and correlation of chemokine and matrix proteins

**Fig. 3:**
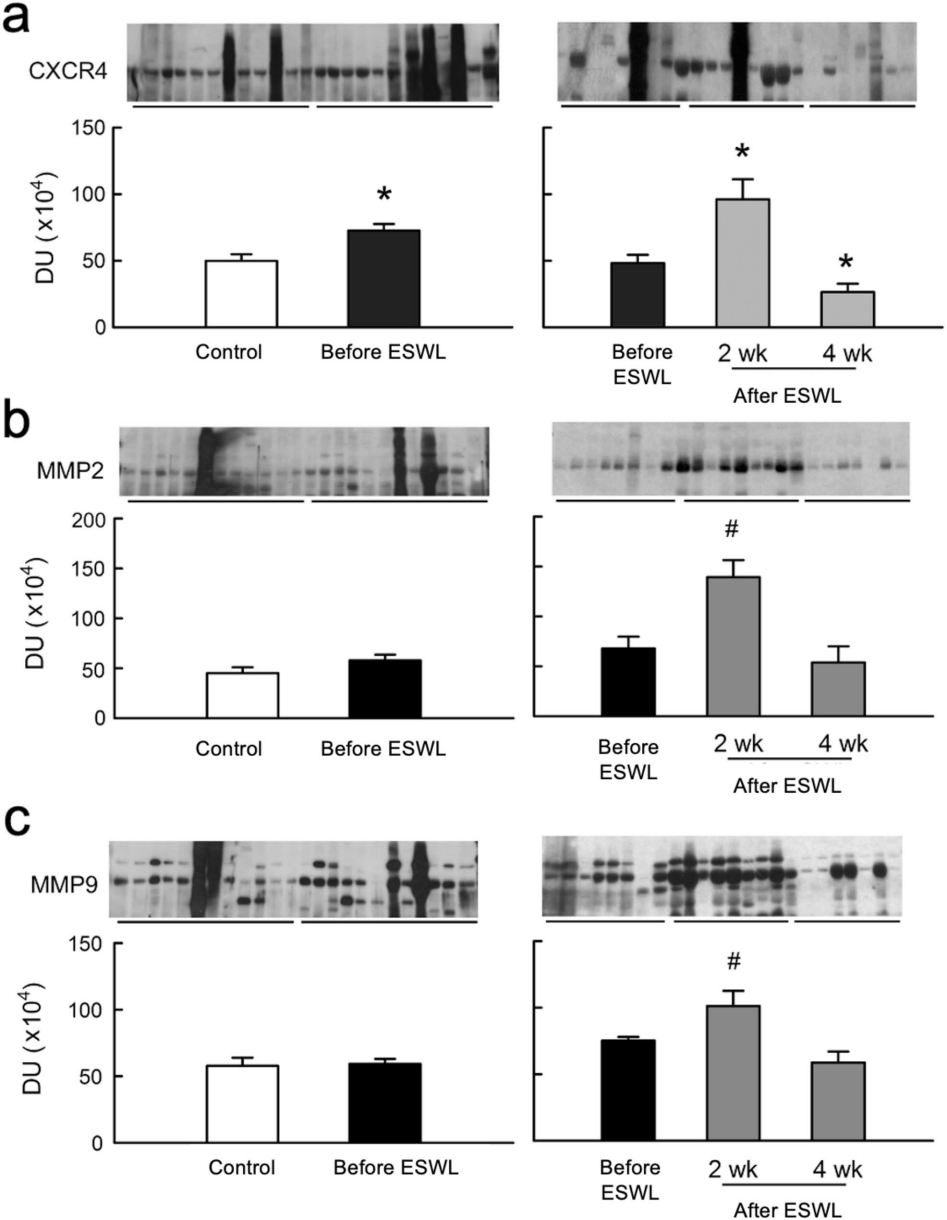
Changes in urinary expression of chemokine and matrix proteins. (**a**) Urinary CXCR4 (40 kDa) levels were significantly higher in stone patients before ESWL compared to controls. Following ESWL, CXCR4 levels significantly increased compared to pre-ESWL levels in stone patients and dropped below pre-ESWL level at 4 week (**b**) MMP2 (Matrix Metalloproteinases 2, 72 kDa) showed no significant different in stone patients before ESWL compared to controls. MMP2 elevated significantly at 2 weeks following ESWL and returned to control level at four weeks. (**c**) MMP9 (Matrix Metalloproteinases 9, 92 kDa) showed no significant different in stone patients before ESWL compared to controls. MMP2 elevated significantly at 2 weeks following ESWL and returned to control level at four weeks

In Fig. 3, urinary CXCR4 levels were significantly higher in stone patients before ESWL compared to controls, while MMP2 and MMP9 showed no significant differences. Following ESWL, CXCR4, MMP2, and MMP9 levels significantly increased. At four weeks post-ESWL, MMP2 and MMP9 levels returned to control levels, whereas CXCR4 levels notably dropped below pre-ESWL and 2-week levels. Table 2 also illustrates the correlation between urinary CXCR4, MMP2, and MMP9 levels and clinical outcomes. Urinary CXCR4 levels correlated significantly with stone size and AP_CaOx_ at all examined intervals, as did MMP2 and MMP9 levels. Temporal changes in correlations with urinary CXCR4 and stone size or AP_CaOx_, and MMP2 (or MMP9) levels and stone size or AP_CaOx_ were significant.

### Analysis of factors influencing stone recurrence after ESWL

Among the 54 enrolled stone patients, 31 were followed up at least once at NTUH from January 2012 to December 2012. Within this group, 15 patients experienced stone recurrence based on imaging studies, yielding a stone recurrence rate of 48.4%. Stratifying these patients based on stone recurrence, factors influencing stone recurrence after ESWL were analyzed using logistic regression. The univariate analysis revealed that factors affecting stone recurrence included urinary OPN at 2 weeks post-ESWL (*p* = 0.024) and APCaOx (*p* = 0.023), urinary SLC26A6 (*p* = 0.046), CXCR4 (*p* = 0.024), and OPN (*p* = 0.010) at 4 weeks post-ESWL(*p* < 0.05). Following multivariate logistic regression (Table 3), only APCaOx (*p* = 0.019) and urinary OPN (*p* = 0.047) at 4 weeks post-ESWL retained significant influence on stone recurrence.

**Table 3:** Univariate and multivariate analysis of factors influencing stone recurrence after ESWL treatment

Parameters	Univariate			Multivariate		
	OR	95% CI	*p* value	OR	95% CI	*p* value
**2 weeks post- ESWL**						
αGST	4.58	0 0.99–21.12	0.051			
πGST	4.40	0.98–19.85	0.054			
AP _CaOx_	4.58	0.99–21.12	0.051			
MDA	3.54	0.78–16.03	0.102			
HA	3.33	0.76–14.58	0.110			
NPC	0.52	0.12–2.17	0.368			
NRP	3.33	0.76–14.58	0.110			
SLC26A6	4.58	0.99–21.12	0.051			
CXCR4	4.40	0.98–19.85	0.054			
MMP2	3.3	0.75–14.47	0.113			
MMP9	4.4	0.98–19.85	0.054			
OPN	6.05	1.27–28.73	0.024*	18.61	0.81–426.9	0.067
THP	0.86	0.21–3.58	0.833			
**4 weeks post-ESWL**						
αGST	0.89	0.22–3.67	0.870			
πGST	0.52	0.12–2.17	0.368			
AP _CaOx_	60.7	2.58–659.3	0.023*	36.53	1.81–737.4	0.019*
MDA	1.56	0.36–6.69	0.553			
HA	0.89	0.22–3.67	0.870			
NPC	1.50	0.36–6.23	0.577			
NRP	2.50	0.59–10.62	0.214			
SLC26A6	5.14	1.03–25.60	0.046	3.33	0.27–41.92	0.352
CXCR4	6.05	1.27–28.73	0.024	6.81	0.41–115.7	0.184
MMP2	0.88	0.21–3.59	0.853			
MMP9	0.88	0.21–3.59	0.853			
OPN	8.67	1.66–45.21	0.010*	13.55	1.75–243.5	0.047*
THP	2.57	0.60–11.06	0.204			

Abbreviations: ESWL, extracorporeal shock wave lithotripsy; OR, odds ratio; CI, confidence interval; APCaOx, ion-activity product of calcium oxalate; MDA, malondialdehyde; GST, glutathione S-transferase; HA, hyaluronic acid; NPC, nuclear pore complex p62; NRP, nucleolin-related protein; OPN, osteopontin; THP, Tamm-Horsfall protein; SLC26A6, solute carrier family 26 member 6 (oxalate transporter); CXCR4, C-X-C chemokine receptor type 4; MMP, matrix metalloproteinase; πGST, pi-glutathione S-transferase; αGST, alpha-glutathione S-transferase

## Discussion

The major findings of our study reveal significant insights as follows: despite successful stone clearance with ESWL, stone patients still exhibit hyperoxaluria and hypocitraturia, leading to elevated urinary APCaOx, a crucial factor driving CaOx crystal formation. Secondly, tubular enzymuria and urinary lipid peroxides remain high post-ESWL treatment, indicating persistent biochemical abnormalities. Additionally, there’s an increased risk of CaOx crystal formation two weeks post-ESWL, evident from elevated urinary excretion of NRP, SLC26A6, CXCR4, MMP2, and MMP9 proteins, potentially promoting crystal attachment and deposition. Finally, increased urinary APCaOx and OPN levels four weeks post-ESWL may indicate urinary stone recurrence.

It is important to acknowledge that kidney stone formation is a multifactorial process involving distinct but potentially coexisting mechanisms. In particular, Randall’s plaques—calcium phosphate deposits within the papillary interstitium—may serve as sites for calcium oxalate stone overgrowth once exposed to urine. At the same time, crystal adhesion to injured tubular epithelial cells, often driven by oxidative stress or altered urinary biochemistry, may also contribute to stone retention. Although our study did not directly evaluate papillary morphology, the urinary biomarkers examined may reflect underlying systemic and renal conditions involved in both interstitial and epithelial pathways. This highlights the relevance of integrating biomarker profiles with mechanistic insights into stone pathogenesis.

Crystals could bind to the renal tubular epithelium under specific conditions [10]. This can occur due to various factors, including the presence of urinary macromolecules (UMM) like THP [19], luminal membrane molecules, such as HA and OPN [21], and surface-associated proteins like NRP [7]. Oxalate binding proteins like NPC are thought to regulate crystallization in hyperoxaluric conditions [6]. SLC26A6 may also modulate CaOx crystal formation by affecting oxalate absorption [22]. Overproduction of CXCR4 and MMP can enhance CaOx crystal aggregation by digesting matrix and regulating inflammation [23]. Organic matter is now recognized as crucial in the mineral phase control of nephrolithiasis [8]. There’s debate whether these substances inhibit mineralization but become overwhelmed by excess crystal formation, or if they associate with the mineral phase through changing interactions [8].

**Effects of urinary stone on crystal-related proteins.**

In this study, urinary levels of NPC, HA, and CXCR4 were significantly elevated before stone treatment, implying a potential relationship with crystal formation in urolithiasis patients. NPC expression may result from oxalate-induced oxidative stress, with its binding activity correlated with lipid peroxidation [24, 25]. Urinary NPC levels increased before treatment and notably decreased post-stone removal, suggesting a correlation with stone presence.

HA, found in renal tubules, is associated with renal tissue damage and may contribute to stone development [26]. Urinary HA levels gradually decreased after ESWL treatment, possibly indicating reduced stress-induced HA production. However, high urinary HA levels post-treatment may still pose a risk for stone recurrence, though not significantly associated in this study. The pathogenesis of nephrolithiasis involves crystal deposition in renal tubules, interstitial plaque overgrowth, with CXCR4 potentially playing a role in monocyte infiltration and renal tissue damage [27, 28]. Decreased urinary CXCR4 expression post-ESWL may indicate reduced monocyte infiltration and subsequent reduced tissue damage.

### Effects of ESWL treatment on urinary parameters

In this study, urinary NRP, SLC26A6, OPN, CXCR4, MMP2, and MMP9 increased at 2-week post-ESWL, with SLC26A6 and OPN remaining high at 4-week post-ESWL.

ESWL is a safe and effective treatment modality for renal and ureteral stone [29], albeit with potential trauma-related complications [29]. Subcapsular hematoma, observed post-ESWL, may explain the significant increase in MMP2 and MMP9 at 2 weeks, which returned to normal at 4 weeks [30]. Urinary NRP expression increased only at 2 weeks post-ESWL, possibly due to increased expression induced by ESWL treatment, consistent with cell culture findings [7]. The positive correlation between urinary SLC26A6 and αGST levels suggests a protective role of SLC26A6, despite no significant decrease in urinary oxalate levels post-ESWL. Increased OPN expression post-ESWL may reflect renal parenchymal injury, potentially contributing to tissue remodeling and inhibiting CaOx crystal formation [31, 32].

### Mechanisms contribute to elevated urinary crystal-related molecules and inflammatory chemokines

An animal study demonstrated increased urinary lipid peroxidation after 7 days of hyperoxaluria induction [33], while macrophage infiltration and renal interstitium widening was observed after 21 days, becoming more prominent by day 42 [28]. This suggests that leukocyte infiltration may not correlate strongly with lipid peroxidation in the kidney. In our study, urinary CXCR4 and MMPs levels correlated positively with stone size and AP_CaOx_, rather than urinary MDA levels, consistent with findings from a hyperoxaluric rat model [28].

Animal studies have shown that hyperoxaluria increases free radical productions in the kidney, potentially inducing apoptosis in renal tubule epithelial cells and enhancing the expression of attachment molecules, facilitating calcium-containing crystal attachment [28, 34]. Deposition of calcium-containing crystals in the kidney coincides with leukocyte infiltration, suggesting an inflammatory response. Clinical studies have linked nephrolithiasis with increased renal tubular damage and upregulation of inflammatory mediators like MCP-1 and interleukin-6 [35, 36].

Hydroxyapatite crystals induce oxidative stress and upregulate inflammatory mediators [37]. While CXCR4 may regulate inflammation and facilitate monocyte infiltration, MMPs play roles in ECM accumulation and degradation of the basement membrane [23]. MMP-2 and MMP-9 could compromise renal parenchyma integrity [23], leading to crystal attachment and growth in tubular lumen. Further experiments are needed to validate this hypothesis.

### Urinary OPN level and AP_CaOx_ at 4-week post-ESWL are associated with stone recurrence

No single theory fully explains kidney stone disease due to its multifactorial nature. Our study proposes that elevated urinary levels of HA, NPC, NRP, CXCR4, MMP-2, and MMP-9, or reduced urinary levels of OPN and SLC26A6 four weeks post-ESWL may indicate multiple risk factors for crystal formation and adherence in stone patients.

Comparison between recurrent and non-recurrent patients after 5 years revealed significant differences in urinary OPN and AP_CaOx_ level at 4-week post-ESWL, suggesting potential indicators for stone recurrence surveillance. However, further validation in large cohort studies is necessary before clinical application.

In summary, our long-term prospective study assessed clinical parameters and crystal-related proteins in stone patients post-ESWL. Before SWL, NPC, HA, and CXCR4 were elevated compared to controls. Post-ESWL at 2 weeks, all markers increased except THP, and at 4 weeks, only SCL26A6 and OPN remained elevated. HA, NPC, NRP, and SLC26A6 correlated with MDA, while CXCR4 and MMPs correlated with stone size and APCaOX. Stratification by stone recurrence highlighted increased OPN and APCaOx at 4 weeks post-ESWL in the recurrent group, suggesting potential stone recurrence indicators.

### Study limitations and future directions

One limitation of our study is the incomplete follow-up, as only 31 out of 54 patients were evaluated at 5 years, which may introduce selection bias. The modest sample size also limits the statistical power to detect subtle associations. Nonetheless, our study was exploratory in nature, aiming to identify potential trends and biologically meaningful patterns rather than to establish definitive conclusions. The associations observed—particularly those involving urinary OPN and APCaOx—are supported by previous studies and warrant further validation in larger, prospective cohorts to clarify their clinical relevance.

Furthermore, we did not capture detailed longitudinal data regarding the development of new comorbidities, changes in medications, or lifestyle modifications over the 5-year follow-up period, all of which may have influenced stone recurrence. Lastly, our study did not include direct evaluation of Randall’s plaque burden via endoscopic or histopathologic assessments. Therefore, while urinary biomarkers such as OPN and SLC26A6 may reflect stone formation risk, their specific association with plaque formation remains to be established. Future studies incorporating comprehensive papillary morphological assessment alongside standardized urinary biomarker surveillance and longitudinal clinical data collection will provide a more robust understanding of stone recurrence mechanisms.

## Conclusion

This study highlights that despite successful stone removal via ESWL, patients exhibited persistent biochemical abnormalities, including elevated APCaOx, urinary lipid peroxides, and tubular enzymuria, which are linked to an increased risk of CaOx crystal formation. Elevated urinary proteins such as NRP, SLC26A6, and OPN post-ESWL are associated with stone recurrence. Elevated OPN and APCaOx at 4 weeks post-ESWL were significant predictors of recurrence, emphasizing the importance of monitoring these biomarkers for personalized management of stone disease.

## Data Availability

The data that support the findings of this study are not openly available due to reasons of sensitivity and are available from the corresponding author upon reasonable request.

## Abbreviations

CaOx: Calcium Oxalate
ESWL: Extracorporeal Shock Wave Lithotripsy
NRP: Nucleolin-Related Protein
NPC: Nuclear Pore Complex p62
HA: Hyaluronic Acid
SLC26A6: Solute Carrier Family 26 Member 6 (Oxalate Transporter)
CXCR4: C-X-C Chemokine Receptor Type 4
OPN: Osteopontin
THP: Tamm-Horsfall Protein
MMPs: Matrix Metalloproteinases
APCaOx: Ion-Activity Product of Calcium Oxalate
MDA: Malondialdehyde
eGFR: Estimated Glomerular Filtration Rate
αGST: Alpha Glutathione S-Transferase
πGST: Pi Glutathione S-Transferase
ANOVA: Analysis of Variance
GLM: Generalized Linear Model
SD: Standard Deviation
PBS: Phosphate-Buffered Saline
HRP: Horseradish Peroxidase
SDS: Sodium Dodecyl Sulfate
PVDF: Polyvinylidene Difluoride
MCP-1: Monocyte Chemoattractant Protein-1
ECM: Extracellular Matrix
IL-6: Interleukin-6
